# Gecko adhesion based sea star crawler robot

**DOI:** 10.3389/frobt.2023.1209202

**Published:** 2023-07-04

**Authors:** Sampada Acharya, Peter Roberts, Tejas Rane, Raghav Singhal, Peize Hong, Viraj Ranade, Carmel Majidi, Victoria Webster-Wood, B. Reeja-Jayan

**Affiliations:** ^1^ Far-from-equilibrium Materials Laboratory, Department of Mechanical Engineering, Carnegie Mellon University, Pittsburgh, PA, United States; ^2^ Soft Machines Lab, Department of Mechanical Engineering, Carnegie Mellon University, Pittsburgh, PA, United States; ^3^ Department of Mechanical Engineering, Carnegie Mellon University, Pittsburgh, PA, United States; ^4^ Department of Biomedical Engineering, Carnegie Mellon University, Pittsburgh, PA, United States; ^5^ The CMU B.O.R.G, Department of Mechanical Engineering, Carnegie Mellon University, Pittsburgh, PA, United States

**Keywords:** sea star, gecko, bio-inspired robots, soft-robots, gecko adhesion

## Abstract

Over the years, efforts in bioinspired soft robotics have led to mobile systems that emulate features of natural animal locomotion. This includes combining mechanisms from multiple organisms to further improve movement. In this work, we seek to improve locomotion in soft, amphibious robots by combining two independent mechanisms: sea star locomotion gait and gecko adhesion. Specifically, we present a sea star-inspired robot with a gecko-inspired adhesive surface that is able to crawl on a variety of surfaces. It is composed of soft and stretchable elastomer and has five limbs that are powered with pneumatic actuation. The gecko-inspired adhesion provides additional grip on wet and dry surfaces, thus enabling the robot to climb on 25° slopes and hold on statically to 51° slopes.

## 1 Introduction

Research in underwater robot locomotion has increased in the last few decades, achieving a successful interaction with the environment for movement-based, control-intensive operations. Tasks such as deep-sea exploration, picking and placing large and heavy objects, pipeline inspection and maintenance and extraction of mineral resources are some of the areas where underwater robots are extensively used (Neira et al., 2021; Wang and Cui, 2021). Most of these underwater robots have rigid bodies and are actuated with electrical motors or hydraulic circuits (Neira et al., 2021), which satisfy the mobility and dexterity requirements for the previously mentioned tasks. However, other relevant underwater tasks are still done manually and require a more flexible gait in order to be done accurately, such as biological sample gathering, archaeological exploration or underwater exploration of otherwise inaccessible areas (Wang and Cui, 2021). These tasks require a soft, flexible robot capable of maneuvering across different surfaces underwater.

Marine creatures have evolved multiple gait and motion techniques along with flexible structures, that allow them to move in different and efficient ways (Gemmell et al., 2015; Mo et al., 2020; Wereley, 2021). These techniques have been widely studied and have served as inspiration for different kinds of underwater mobile soft robots like swimmers (Patterson et al., 2020; Ulloa et al., 2020; Shen et al., 2020; Huang et al. (2022; 2021), walkers (Corucci et al., 2015; Wu et al., 2021), crawlers (Tan et al., 2021) or a combination of these gaits like swimming and crawling (Arienti et al., 2013; Giorgio-Serchi et al., 2017).

**FIGURE 1:**
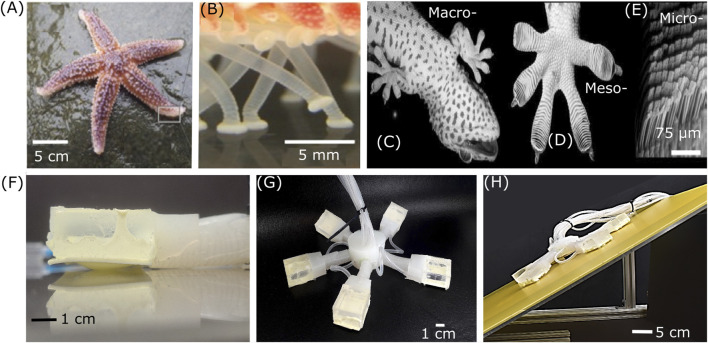
**(A–E)** Bioinspiration Images **(A)** Common sea star (*Asterias rubens*) (Image adapted from Heydari et al. (2020) (source: Shutterstock) with permission conveyed through Copyright Clearance Center, Inc.). **(B)** Tube feet of the sea star (Image adapted from Heydari et al. (2020), (source: Symbiotic Service, San Diego) with permission conveyed through Copyright Clearance Center, Inc.). **(C)** Ventral view of a tokay gecko *(Gekko gecko)*. **(D)** Tokay gecko foot, showing array of setae-bearing scansors. **(E)** Microscale array of setae (Images adapted from (Autumn and Gravish, 2008) with permission conveyed through Copyright Clearance Center, Inc.).**(F)** Robot’s limb with gecko patch actuated. **(G)** Gecko Adhesion Based Sea Star (GASS) Crawler Robot. **(H)** GASS robot climbing a 25° slope.

Inspired by the sea star locomotion (Figure 1A) which includes crawling and grounded bouncing (Heydari et al., 2020), many attempts have been made to create a sea star crawler (Youssef et al., 2022). However, these studies have not taken into consideration the tube feet (*podia*) (Figure 1B), which are organs found in echinoderms that provide sensing capabilities, contribute to feeding, allow the sea star to grip onto various surfaces and contribute to its locomotion (Sngster and Brown, 1972; Heydari et al., 2020). Recent studies have attempted to incorporate tube feet into sea star-inspired robots using magnetic fields for actuation (Yang et al., 2021) and adhesion (Bell et al., 2018).

An alternative to using tube feet is to incorporate microstructures based on gecko adhesion. Gecko lizards (Figure 1C) have nano-fibrillar structures on their feet (Figures 1D, E) that allow them to adhere to multiple types of surfaces without any tackiness by means of weak van der Waals forces, thus enabling them to walk vertically and even upside down (Tian et al., 2006). Inspired by this mechanism, many adhesive surfaces have been fabricated with varying degrees of abstraction or biomimickry, such as mushroom-shaped microstructures (Song et al., 2013; Li et al., 2020; Tian et al., 2020), microfabricated wedges (Parness et al., 2009; Glick et al., 2018; Simaite et al., 2018), and pillar shaped microstructures (Mengüç et al., 2012; Kizilkan and Gorb, 2018) for applications in soft grippers (Cauligi et al., 2020; Song and Sitti, 2014; Glick et al., 2018; Song et al., 2017) and wall climbing robots (Menon et al., 2004; Aksak et al., 2008).

To improve locomotion of sea star-inspired robots, in this work, we present a soft sea star robot that combines limb motion and surface adhesion for crawling. Our Gecko Adhesion Based Sea Star (GASS) Crawler Robot is a pneumatically actuated soft robot that combines sea star-inspired limb extension and gecko-inspired adhesion to be able to crawl on a variety of materials, dry and wet surfaces, and even sloped surfaces (Figures 1F–H). In this paper, we present the design, fabrication, and characterization of the GASS Crawler Robot, including the fabrication and integration of gecko-inspired adhesive foot patches to mimic sea star tube feet and soft extension actuators for each leg. We tested the foot patches on glass, acrylic, and stainless steel surfaces under dry and wet conditions with and without adhesion. Activation of adhesive patches reduced slipping on sloped surfaces. Furthermore, we assessed the performance of the GASS crawler robot on an acrylic surface under wet and dry conditions. Activation of the gecko-inspired adhesive feet significantly improved locomotion performance under all experimentally tested conditions.

## 2 Design and fabrication

### 2.1 Design overview

The robot consists of a five-limb soft sea star-inspired design with amphibious crawling capacity. To mimic the sea star crawling motion, the robot was programmed with two forms of actuation—one that could adhere and detach the robot from the surface and one that could make the robot move forward. In order to pull/push the robot to achieve locomotion, each limb consisted of a combination of these two forms of actuation. The tip of the limb consisted of a pneumatically actuated expandable gecko patch. When air was pumped into the actuator, the patch inflated, touching the surface below it. The internal air pressure exerted enough pressure for the patch to attach to the surface, giving the robot the desired adhesion. When the internal air pressure was released, the patch was detached from the surface.

For the adhesion actuator to have a forward motion while detached, a linear actuator was included in the limb. This actuator consisted of a pneumatically actuated stretchable cylinder wrapped with a stainless steel wire mesh. This wire mesh constrained the cylinder’s radius. When air was pumped into the cylinder, its expansion was mostly linear along the axis of the leg, giving the limb the ability to extend itself in a forward direction.

The combination of these two motions gave each limb the ability to extend and attach itself to a specific point and then pull the robot towards it or push the robot against it, enabling a crawling motion for the robot. The limbs were connected to the robot’s main body with uniform angular distribution mimicking the structure of a five-limbed sea star (Figure 1G), mimicking with the most common species of sea star (Rahman et al., 2018).

### 2.2 Fabrication

#### 2.2.1 Gecko patch fabrication

**FIGURE 2:**
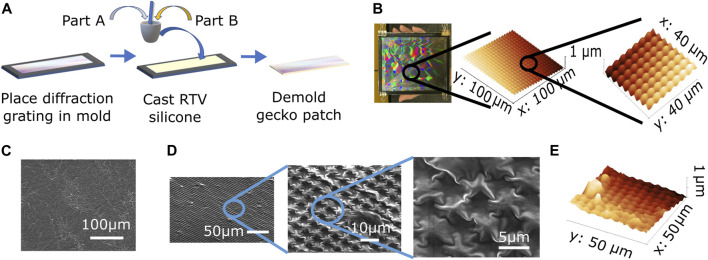
**(A)** Gecko adhesive patch fabrication process. **(B)** Optical and atomic force microscopy (AFM) images of diffraction grating. **(C)** Scanning electron microscope (SEM) image of unpatterned silicone surface. **(D)** SEM images of patterned silicone surface. **(E)** AFM image of patterned silicone surface.

A multi-step fabrication process was implemented to create the soft, gecko-inspired adhesive patch in a cost-effective, fast and facile manner. First, a two-part mold having dimensions 150 × 100 × 0.7 mm was laser-cut from an acrylic sheet. Then, the diffraction grating (Rainbow Symphony^®^, 500 lines/cm) was attached to the bottom half of the mold and secured using the top half. Clamps were attached to the sides of the mold to secure it in place. Next, a two-part, high strength room temperature vulcanizing (RTV) silicone from Illuseffects^®^ was mixed in the concentration ratio of 10:1. The trapped air bubbles were removed by degassing under vacuum to remove any air bubbles. Following that, the silicone was poured into the mold and degassed again under vacuum for 30 min to remove the air bubbles. It was then allowed to cure for 24 h at room temperature, after which it was manually peeled from the mold to obtain the gecko patch (Figure 2A).

The micro-pattern of hemispheres on the diffraction grating was confirmed using atomic force microscopy (AFM). The height of each hemisphere was 1 *μm* and the radius at the base was 1.87 *μm* (Figure 2B). The scanning electron microscope and atomic force microscope images of plain silicone (Figure 2C) and gecko patch (Figures 2D, E) showed that the pattern on the diffraction grating was successfully imprinted onto the silicone surface, thereby creating the gecko patch. The RTV silicone flowed into the gaps between the hemispheres resulting in an approximate hemispherical structure of height 1 *μm* and base radius 1.87 *μm*.

To evaluate the hydrophobic characteristics of the gecko patches, contact angle measurements were taken across 3 different samples, obtaining an average angle of 114.239° with a standard deviation of 5.89°.

#### 2.2.2 Soft linear actuator fabrication

The linear actuator was designed based on the earthworm-inspired soft robot presented in Calderon et al. (2016). Its fabrication was done in two steps. First, a soft, hollow cylinder was made using Ecoflex^®^ 00-10. The soft cylinder was then wrapped with a stainless steel wire mesh. Its purpose was to constrain the external radius of the actuator so that when the air was pumped inside the cylinder, it expanded only in the longitudinal direction while maintaining an approximately constant radial dimension.

**FIGURE 3:**
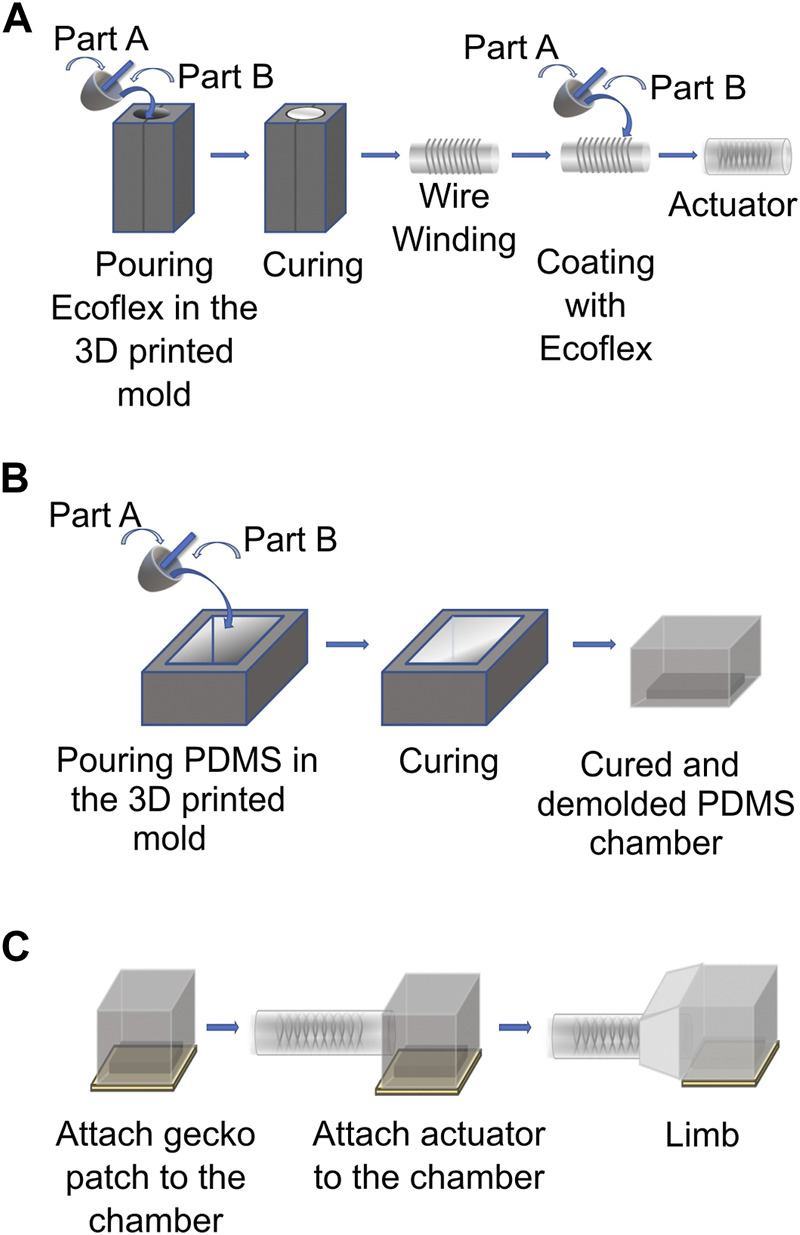
**(A)** Fabrication steps for the soft actuator. **(B)** Fabrication steps for the robot foot. **(C)** Fabrication process of the soft limb.

For the fabrication process, a mold was 3D printed using Grey Resin from Formlabs^®^ in a Formlabs^®^ Form3 3D printer. A 1:1 concentration of Ecoflex^®^ 00-10 was mixed and poured in the mold and degassed under vacuum for 30-40 min to extract all the trapped air bubbles. Following this, the mold was placed in an oven at 80°C for 6 h to cure the soft cylinder. Once fully cured, the cylinder was extracted from the mold and, using a 0.5 mm diameter stainless steel wire, a double helix wire mesh was manually wrapped around the cylinder with a 2–3 mm distance between the turns. The mesh-wrapped soft cylinder was then manually covered with a thin layer of Ecoflex^®^ 00-10 to seal it. Next, a 5 mm diameter silicone tube was inserted in the cylinder and sealed with Ecoflex^®^ 00-10. The cylinder was then inserted in another 3D printed mold and degassed under vacuum for 15 min to remove any air bubbles and was then put in an oven at 80°C for 4 h to cure. Once cured, it was demolded to obtain the soft pneumatic linear actuator (Figure 3A).

#### 2.2.3 Robot foot fabrication

The distal end (“foot”) of the soft robot limb was fabricated as follows: first, a hollow polydimethylsiloxane (PDMS) chamber was fabricated by mixing Sylgard^®^ 184 in a 10:1 concentration ratio of part A to part B. The mixed PDMS was then degassed in a Thinky^®^ mixer in the “degas” mode for 6 min. The PDMS was then poured into a 3D printed Formlabs^®^ Grey Resin mold and degassed again under vacuum for 30 min. After this, it was cured in an oven at 80°C for 24 h and was demolded (Figure 3B).

#### 2.2.4 Limb fabrication

To make the soft limb, the chamber fabricated as described in Section 2.2.3 was attached to a 4 × 4 *cm* ^2^ gecko patch using RTV silicone from Illuseffects^®^. To attach the linear actuator and the gecko chamber and maintain the mechanical properties needed for a soft and stretchable limb, Ecoflex^®^ 00-10 was used in a 1:1 concentration since it was also used for the linear actuator and it has shown good adhesion to PDMS Yu et al. (2015).

A mold was 3D printed using Grey Resin from Formlabs^®^ in a Formlabs^®^ Form3 3D printer and it was used to hold both the soft linear actuator and the gecko chamber in place, and it was filled with Ecoflex^®^ 00-10 to bond them together (Figure 3C).

### 2.3 Robot controller

**FIGURE 4:**
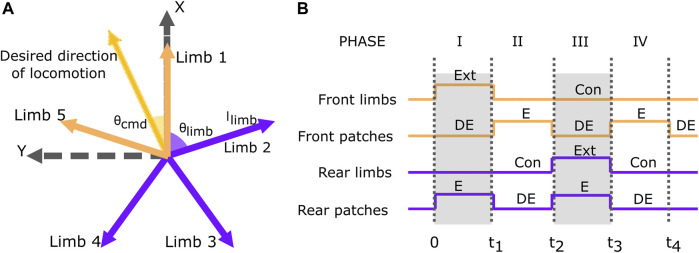
**(A)** Schematic vector diagram of limbs for movement of GASS Robot. Here *l*_*limb*_ and *θ*_*limb*_ denote the magnitude and angle of the movement vector for the limb, and *θ*_*cmd*_ denotes the commanded desired direction of movement of the robot. **(B)** The timing diagram for the basic crawling gait. Here, “Ext” and “Con” refer to extension and contraction of soft limbs respectively, and “E” and “DE” refer to engagement and disengagement of the gecko patch with the surface.

The crawling motion of the robot was visualized as the linear combination of five movement vectors corresponding to each limb in the x-y plane. The desired direction of the robot movement determined the magnitude of the movement vector for each limb following the control law mentioned in Eq. 1 and shown in Figure 4A.

$$l_{\mathrm{limb}}=l_{\max}+\cos\left(\theta_{\mathrm{cmd}}-\theta_{\mathrm{limb}}\right)$$ (1)

Here, *l*_*limb*_ denotes the magnitude of each limb’s extension where *limb* = 1*to*5 while *θ*_*limb*_ denotes the angle of the limb, which is fixed related to the position of the limb in the robot’s body. *θ*_*cmd*_ denotes the commanded desired direction of movement of the robot, and *l*_*max*_ denotes the maximum magnitude of movement vector, which is a constant determined by the maximum extension for the limbs. Eq. 1 finally determines the required limb extension for each of the five limbs according to the desired direction of motion for the robot.

**FIGURE 5:**
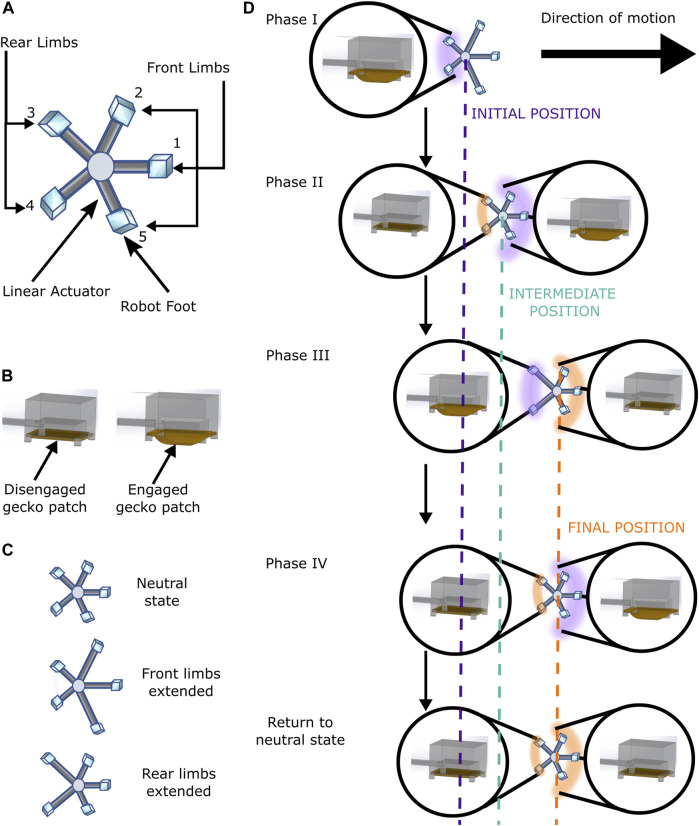
**(A)** Schematic of GASS robot. **(B)** Disengaged (left) and engaged (right) position of the gecko patch on the robot foot. **(C)** States of linear actuators. **(D)** Sequence of GASS robot motion phases for one cycle of motion.

Based on the robot’s desired direction of motion, the movement vectors *l*_*limb*_ were calculated for each limb to execute the required motion and the robot followed a basic crawling gait for locomotion (Figure 4B). To explain the crawling gait cycle, the limbs in the direction of robot motion and the corresponding gecko adhesion patches were called *front limbs* and *front patches*. The remaining limbs and gecko adhesion patches were called *rear limbs* and *rear patches* (Figure 5A). The crawling gait of the robot involves four motions for each locomotion cycle (Figures 5B, C, D). In the first phase, the *front limbs* were extended according to the *l*_*front*_ _*limb*_ calculated by the controller, and the *rear patches* were engaged to start the movement. The second phase was the “pull phase”, where the *front patches* were engaged, *rear patches* were disengaged, and the *front limbs* were contracted to pull the robot in the direction of motion. The third phase was the “push phase”, where the *front patches* were disengaged, *rear patches* were engaged, and the *rear limbs* were extended to push the robot in the direction of motion, with a magnitude *l*_*rear*_ _*limb*_ calculated by the controller. In the fourth phase, the *front patches* were engaged, the *rear patches* were disengaged and the *rear limbs* were contracted, and finally, the *front patches* were disengaged to end the gait cycle.

The limbs and the gecko adhesion patches were operated in a sequential manner, as mentioned above, by an open-loop controller code, such that the motion of the *front limbs* and *rear limbs* does not affect each other during the gait cycle. Since the control strategy was open-loop, it did not account for the different behaviour of the limbs of the soft robot, even if the commanded input was the same or for any deviation that occurred during the robot’s motion.

## 3 Experimental characterization methods

### 3.1 Limb actuator characterization

In order to test the adhesion performance of the soft limb fabricated as described in Section 2.2, we decided to measure the force needed for the limb to start slipping on glass, acrylic, and stainless steel surfaces in both dry and wet conditions. We dispersed 5 mL of deionized water under the soft limb to test its performance in wet conditions. For each surface and condition, 20 tests were performed.

**FIGURE 6:**
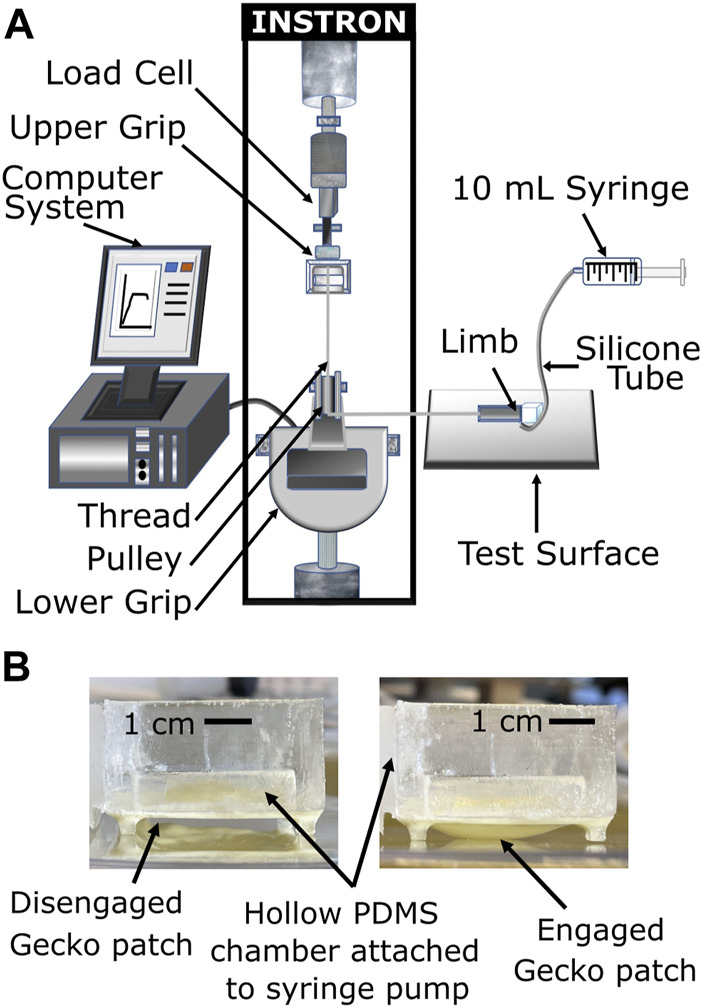
**(A)** Schematic illustration of the limb adhesion test setup. **(B)** Gecko patch in the disengaged state (left) and in the engaged state (right).

The soft limb was attached to one end of an inextensible cotton thread which was looped over a pulley attached to the bottom grip of a Instron^®^ 5969 tensile testing machine. The other end of the thread was fixed into the upper grip of the tensile testing system (Figure 6A). 2 mL of air was then manually pumped into the gecko chamber to inflate the gecko patch and establish adhesion to the surface (Figure 6B). The thread was then pulled at a rate of 0.5 mm/s till the limb started to slip and move backwards. The test ended when the load cell’s displacement reached 20 mm.

### 3.2 Adhesion testing

**FIGURE 7:**
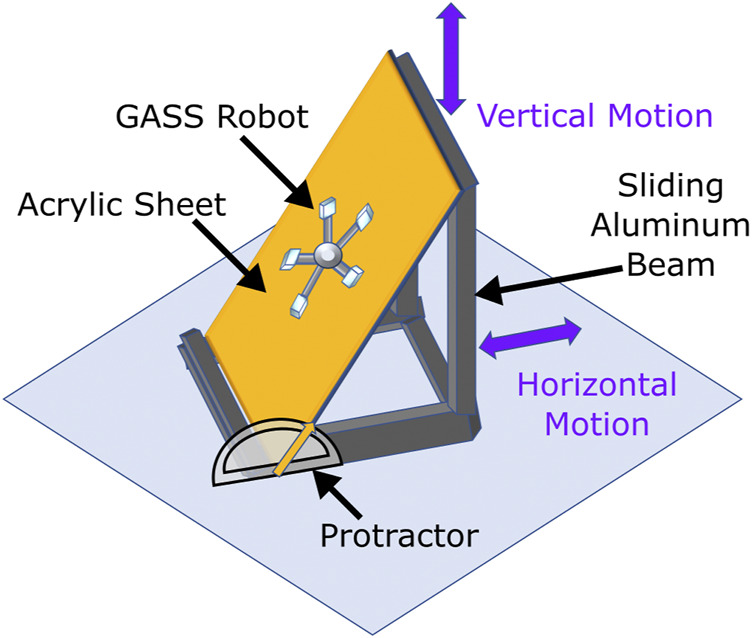
Schematic illustration of static adhesion test measurement setup.

To test the static adhesion of the entire robot, a custom sliding setup was built (Figure 7). The robot was placed on a rigid flat acrylic surface, and all five gecko patches were actuated, ensuring contact and adhesion of its five limbs to the surface. The board was then lifted from one end by sliding the aluminum bars, gradually increasing its slope, while the opposite end was fixed as a pivot while measuring the angle with a protractor (±1° instrumental error) attached to the base. The slope was increased manually until the robot started slipping. The test was repeated 5 times with and without engaging the gecko patches to measure the static adhesion of the robot.

### 3.3 Robot motion testing

To demonstrate the actuators’ functionality, the robot’s ability to crawl was tested on a flat acrylic horizontal surface. The robot’s motion was evaluated according to the limb’s actuation sequence described in Section 2.3. The robot’s continuous motion was evaluated over four cycles of the locomotion controller.

In order to evaluate the robot’s adhesion performance and how the gecko adhesion contributed to the crawling motion, the same test was conducted without actuating the gecko patches, making the robot move only by the action of its limbs extending and contracting. In these tests, the robot controller was programmed so that the gecko patches remained disengaged while maintaining a constant total gait time.

Both test sequences were also conducted on a wet surface to test the effect of gecko adhesion on the robot’s crawling motion in partially flooded areas. The wet surface test was facilitated by pouring 30 mL of deionized water on the acrylic sheet, placing the robot over it, and running the same motion cycles.

The robot’s motion was also evaluated in sloped surfaces, in an upward crawling motion. Using the same set-up for the adhesion test described in Section 3.2 the robot was placed on the surface with all five robot foot actuated to ensure adhesion. The slope angle was manually increased from 0°, at each new angle the control sequence described in Section 2.3 was tested, until the surface reached an angle where the robot showed no forward motion.

## 4 Results

### 4.1 Individual limb

**FIGURE 8:**
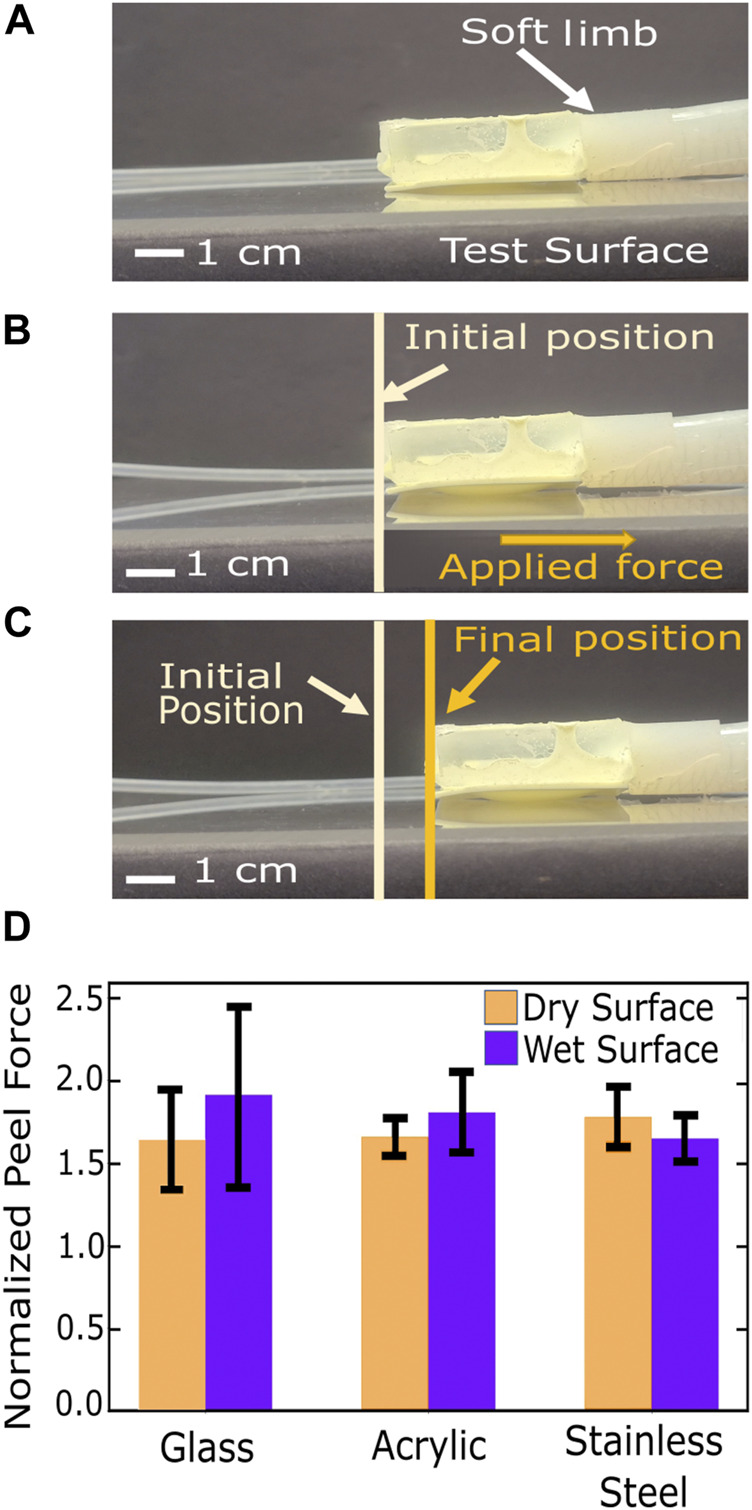
**(A–C)** Images of limb peel test taken from video footage. **(A)** Limb was placed on the test surface **(B)** The gecko patch was inflated with 2 mL of air and the initial position was noted. **(C)** Peel force was applied to the limb (Figure 6A). After a critical slipping force, the limb started slipping. The final position of the limb was noted after the Instron crosshead reached a displacement of 20 mm. **(D)** Limb peel test comparison on different surfaces (glass, acrylic and metal) in both, wet and dry conditions.

The robot limb characterization test was performed as described in Section 3.1 (Figures 8A, B, C). The limb showed the highest adhesion to the dry acrylic surface. Overall, the relative adhesion improvement with the gecko patch activated didn’t show a significant difference across surfaces (*p*-values: 1.7 × 10^−6^, 3.7 × 10^−6^, 5.7 × 10^−4^) (Figure 8D). Thus, all the further tests were performed on an acrylic surface.

### 4.2 Robot motion results

**FIGURE 9:**
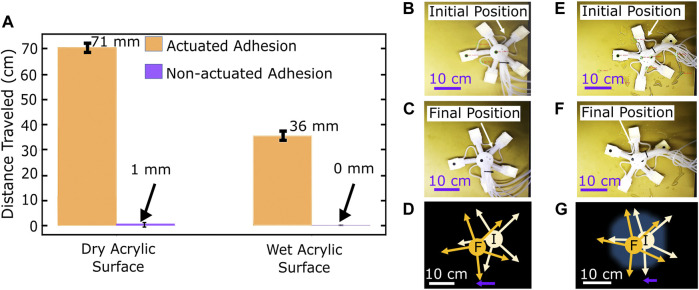
**(A)** Total distance traveled by the robot on dry and wet horizontal acrylic surfaces with and without adhesion in four cycles. **(B)** Initial position of the robot on horizontal, dry acrylic surface, image taken from video footage. **(C)** Final position of the robot on horizontal, dry acrylic surface, image taken from video footage. **(D)** Displacement of robot on horizontal, dry acrylic surface. **(E)** Initial position of the robot on horizontal, wet acrylic surface, image taken from video footage. **(F)** Final position of the robot on horizontal, wet acrylic surface, image taken from video footage. **(G)** Displacement of robot on horizontal, wet acrylic surface.

The motion step cycle, as described in Section 2.3, was executed once to estimate the time required to complete one motion cycle. The robot required 18; to execute one motion cycle and traveled an average horizontal distance of 20 mm (Figure 9D).

As described in Section 3.3 to test the robot’s continuous motion, four cycles were evaluated under different conditions, running each test 5 times on an acrylic surface. On a horizontal dry surface, the whole motion took an average time of 74.6 s, and the robot advanced 70.6 ± 1.7 mm in a straight line, giving the robot an average speed of 0.95 ± 0.02 mm/s. When tested without the gecko adhesion actuated, the robot moved 1.2 ± 0.4 mm, giving it a speed of 0.016 ± 0.005 mm/s. This result confirmed our hypothesis by showing that the use of actuated adhesion improves the robot’s crawling speed by a factor of 59. On the same surface, 30 mL of deionized water was added, and the robot was placed on it to test motion on wet surfaces. A linear motion of 35.6 mm was achieved. When tested without the gecko actuation, the robot only moved 0.1 mm, which can be considered negligible. These results further corroborated the previous conclusion about actuated adhesion improving the robot’s motion.

**FIGURE 10:**
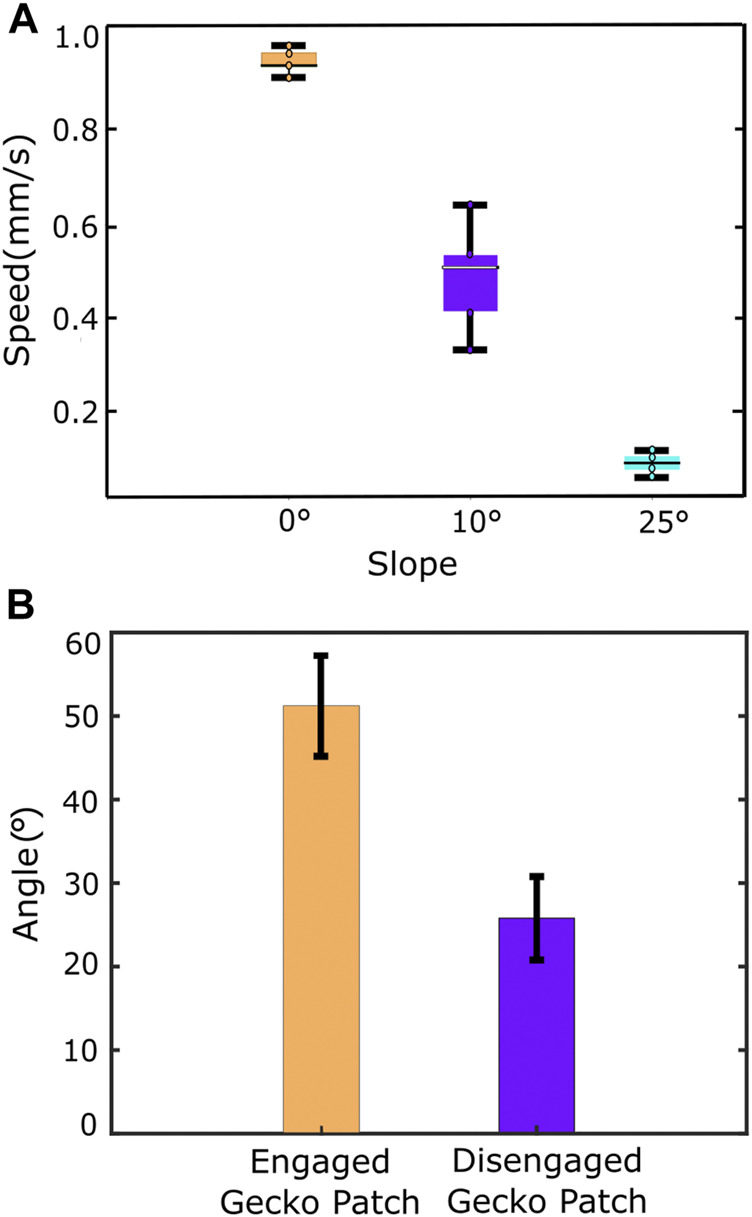
**(A)** Effect of increase in slope on the climbing speed of the robot. **(B)** Static adhesion test results for the robot. Error bars indicate standard deviation.

The robot was able to climb on inclined surfaces, reaching a maximum of 25° ± 1° (error corresponds to the protractor’s instrumental error) for an upward crawling motion, with an average linear displacement of 7.0 ± 1.4 mm for one motion cycle, thus obtaining a climbing speed of 0.38 ± 0.05 mm/s (Figure 10A), errors correspond to the standard deviation of the measurements.

### 4.3 Static robot adhesion

The maximum angle to which the robot stayed adhered to the acrylic surface without slipping was 51° ± 6° on average when the gecko patch was engaged and was 26° ± 5° on average when the gecko patches were disengaged in static condition (Figure 10B), errors correspond to the standard deviation of the measurements. This proves that the gecko patch contributes towards the robot’s ability to stay put on inclined surfaces, and by using the gecko patch, the performance of the robot improved by nearly 2 times.

## 5 Discussion and conclusion

A pneumatically actuated soft sea star-inspired crawling robot was presented, where the use of actuated gecko-inspired adhesion to actively attach and detach itself to the surface improved the robot’s locomotion by a factor of 59 on flat dry surfaces.

The use of actuated gecko-inspired adhesion for locomotion not only gave the robot the ability to adhere to dry surfaces but also to attach itself to wet surfaces and crawl on them, demonstrating the potential for amphibious motion capabilities.

The use of gecko-inspired adhesion was tested for locomotion on inclined surfaces, with the robot effectively crawling up a 25° slope and holding itself statically to slopes reaching up to 51° ± 6°.

The robot’s limb actuator was tested on three different surfaces, where its adhesion was evaluated on wet and dry conditions, obtaining successful results, demonstrating the use of the robot’s limbs on these surfaces.

The GASS Crawler Robot works with an open-loop control system. Sensor data integration needs to be implemented to program a closed-loop control algorithm that can optimize its motion and make the robot follow a trajectory.

Furthermore, the fabrication process involves a number of manual steps that can be perfected for a standardized fabrication method.

All five limbs of the GASS crawler were designed to be identical so that the robot would have pentaradial symmetry. Future designs may include a different number of limbs to test the relation between limb number and mobility.

In addition, different gecko-inspired adhesives can be used to further improve the adhesion capabilities of the robot. With these improvements in fabrication and controls, the GASS crawler can be more robust and capable of faster locomotion speeds. It can also be used on a wider range of surfaces under more variable environmental conditions.

## Data Availability

The raw data supporting the conclusion of this article will be made available by the authors, without undue reservation.
