# Supplementary figures and images for: Gecko adhesion based sea star crawler robot
Source: Front Robot AI. 2023 Jul 4;10:1209202. doi: 10.3389/frobt.2023.1209202 (PMC10352780; doi:10.3389/frobt.2023.1209202)

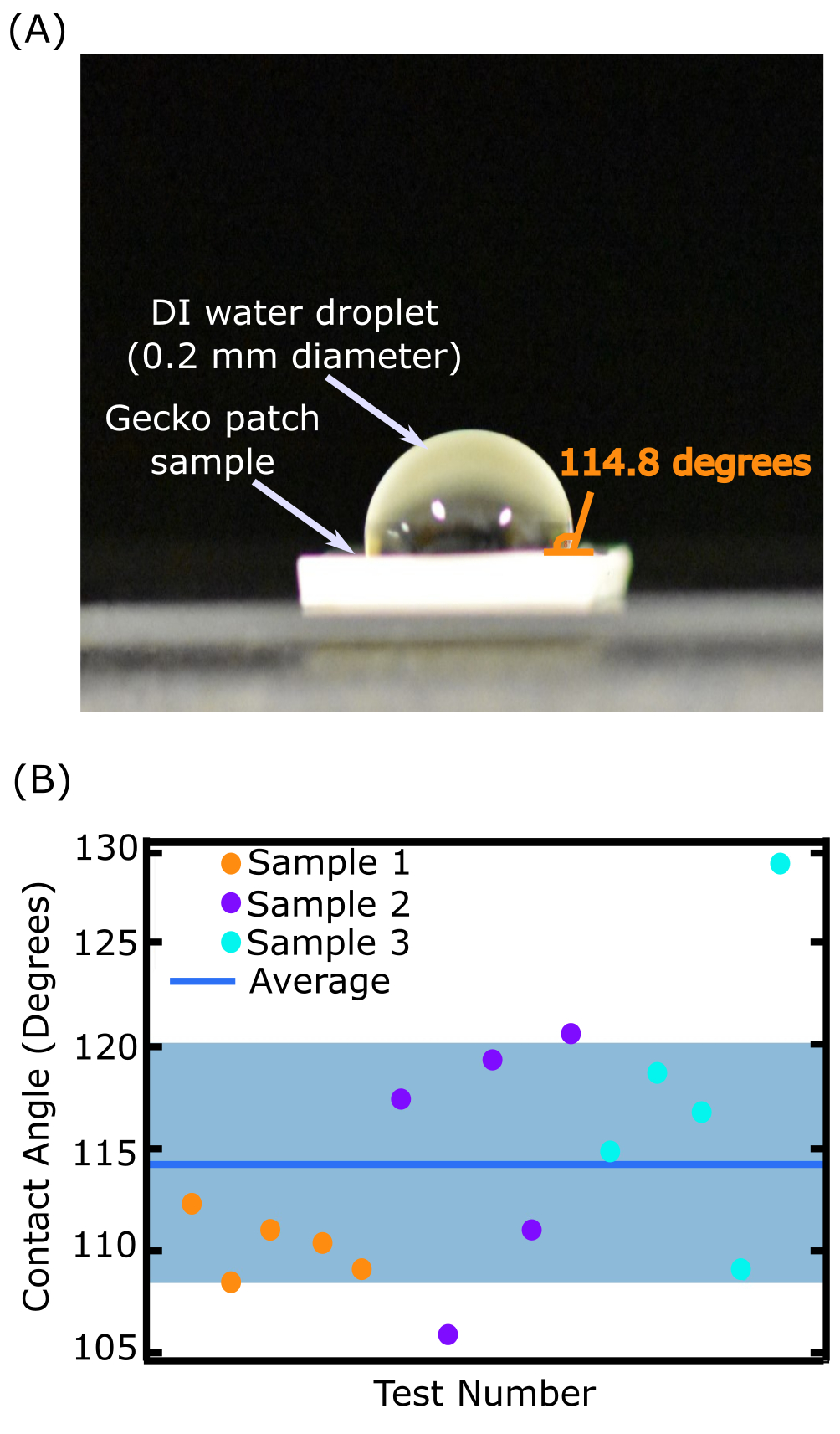

Supplement: Supplementary file 1 [file Image1.JPEG]
