# Supplementary material for: Gecko adhesion based sea star crawler robot
Source: Front Robot AI. 2023 Jul 4;10:1209202. doi: 10.3389/frobt.2023.1209202 (PMC10352780; doi:10.3389/frobt.2023.1209202)
Supplement: Supplementary file 2 [file DataSheet1.PDF]

Supplementary Material

1 SUPPLEMENTARY DATA

1.1 Contact angle measurement

To evaluate the hydrophobic characteristics of the gecko patches fabricated for the GASS robot's feet, we performed contact angle measurements across 3 different samples using the Sessile Drop Method.

Each sample was placed on an optical table with the microtextured side facing upwards. Using a graduated pipet, a 0.2 ml drop of deionized water was placed on the patch's surface. Using a high resolution camera, an image of the drop was taken as seen in figure S1.A. After that, the drop was removed and the surface cleaned with isopropyl alcohol (IPA). This procedure was repeated five times for each sample.

The images were later processed using a Python script for edge detection to determine the tangent line to the droplet at the intersection with the gecko patch's horizontal surface. Then the angle formed between these two lines was obtained. All angle measurements are shown in figure S1.B.

A mean angle of $114.239°$ with a standard deviation of $5.89°$ was obtained accross all samples, satisfying the general rule of being higher than $90°$ for the material to be clasified as hydrophobic.

1.2 Figures

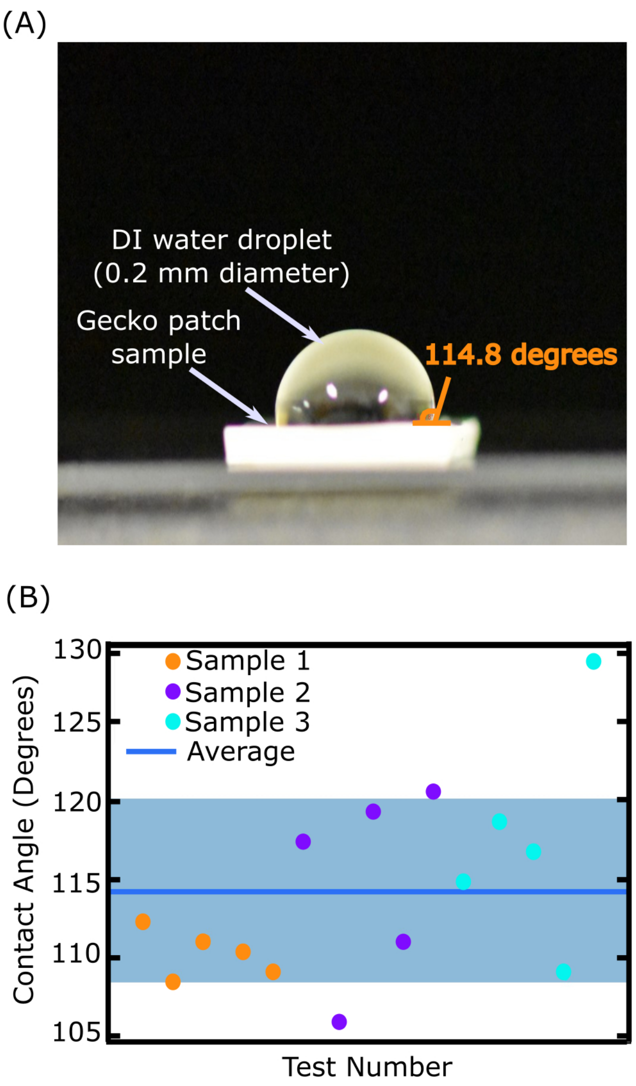

Figure S1. Contact angle measurement. A) Image from one test performed with a 0.2 ml deionized water drop on a gecko patch sample. B) Scatter plot with results obtained from the 3 samples tested
